# Delayed Bone Age and Osteoprotegerin Levels in Pediatric Celiac Disease: A Three-Year Case–Control Study

**DOI:** 10.3390/nu17142295

**Published:** 2025-07-11

**Authors:** Ruzha Pancheva, Yoana Dyankova, Niya Rasheva, Krassimira Koleva, Violeta Iotova, Mariya Dzhogova, Marco Fiore, Miglena Georgieva

**Affiliations:** 1 Department of Hygiene and Epidemiology, Faculty of Public Health, Prof. Dr. Paraskev Stoyanov Medical University, 9000 Varna, Bulgaria; mariadzhogova@gmail.com; 2 Department of Pediatrics, Faculty of Medicine, Prof. Dr. Paraskev Stoyanov Medical University, 9000 Varna, Bulgaria; y.dyankova@abv.bg (Y.D.); niq_rasheva78@abv.bg (N.R.); krasi0617@gmail.com (K.K.); violeta.iotova@mu-varna.bg (V.I.); mgeorgieva7@yahoo.com (M.G.); 3 Institute of Biochemistry and Cell Biology (IBBC-CNR), Department of Sensory Organs, Sapienza University of Rome, 00185 Rome, Italy; marco.fiore@cnr.it

**Keywords:** celiac disease, osteoprotegerin, child, bone age

## Abstract

**Introduction:** Celiac disease (CD) impairs bone development in children through inflammation and nutrient malabsorption. Osteoprotegerin (OPG), a decoy receptor for RANKL, plays a role in bone remodeling and is increasingly recognized as a potential biomarker of bone metabolism and inflammation. However, its clinical significance in pediatric CD remains unclear. **Aim:** To evaluate the relationship between OPG levels, growth parameters, and delayed bone age in children with CD, and to assess OPG’s potential as a biomarker of bone health and disease activity. **Methods:** This three-year case–control study included 146 children: 25 with newly diagnosed CD (Group A), 54 with established CD on a gluten-free diet (Group B), and 67 healthy controls (Group C). Participants underwent clinical, anthropometric, and laboratory assessments at baseline and after 6 months (Groups A and B). OPG and osteocalcin were measured, and bone age was assessed radiologically. Statistical analyses included ANOVA, Spearman’s correlations, and binomial logistic regression. **Results:** OPG levels were highest in newly diagnosed children (Group A), showing a non-significant decrease after gluten-free diet initiation. OPG correlated negatively with age and height in CD patients and controls, and positively with hemoglobin and iron in Group B. Logistic regression revealed no significant predictive value of OPG for delayed bone age, although a trend was observed in Group B (*p* = 0.091). Children in long-term remission exhibited bone maturation patterns similar to healthy peers. **Conclusions:** OPG levels reflect disease activity and growth delay in pediatric CD but lack predictive power for delayed bone age. While OPG may serve as a secondary marker of bone turnover and inflammatory status, it is not suitable as a standalone biomarker for skeletal maturation. These findings highlight the need for integrative biomarker panels to guide bone health monitoring in children with CD.

## 1. Introduction

Celiac disease (CD) is a systemic autoimmune disorder characterized by an aberrant immune response to gluten, a protein found in wheat, barley, and rye, in genetically susceptible individuals [1]. The ingestion of gluten triggers an inflammatory cascade within the small intestine, leading to villous atrophy and crypt hyperplasia, ultimately impairing nutrient absorption [2]. Once believed to be an uncommon condition primarily affecting children and characterized by severe malabsorption and a flattened intestinal lining, CD is now acknowledged as a prevalent autoimmune disorder with a global reach of 1.4%, capable of manifesting at any age with diverse clinical presentations [3,4,5].

Bone health is frequently compromised in individuals with CD, especially during childhood and adolescence, critical periods for skeletal development and the accrual of peak bone mass [6]. The pathophysiology of bone involvement in celiac disease is multifactorial, involving nutrient malabsorption, chronic inflammation, and alterations in the gut microbiome [7]. Malabsorption of key nutrients such as calcium, vitamin D, and vitamin K can directly impair bone mineralization and increase the risk of osteopenia and osteoporosis [8]. The chronic inflammatory milieu characteristic of CD exerts a profound influence on bone metabolism by disrupting the delicate balance between osteoblast-mediated bone formation and osteoclast-mediated bone resorption. Delayed bone maturation is common in pediatric celiac disease, reflecting the impact of chronic inflammation and nutritional deficiencies on the growth plates [9].

Markers of bone health and inflammation, particularly osteoprotegerin (OPG), are increasingly recognized as important in understanding the pathogenesis of bone involvement in CD. OPG acts as a decoy receptor for RANKL, inhibiting osteoclastogenesis and thus bone resorption. In celiac disease, both untreated and treated with a gluten-free diet, alterations in the OPG/RANKL axis and elevated inflammatory cytokines such as IL-6 and IL-18 have been observed, contributing to increased bone turnover and reduced bone mineral density (BMD) [10,11,12,13].

Elevated OPG levels in celiac patients, especially those with persistent low BMD despite a long-term gluten-free diet, may represent a compensatory response to ongoing bone resorption and inflammation but are insufficient to normalize bone health in all patients [10,11]. The persistent imbalance in osteoclastogenesis-regulating factors, including a reduced OPG/RANKL ratio, is associated with lower BMD and increased fracture risk [11]. Additionally, autoantibodies against OPG have been identified in a subset of celiac patients and are independently associated with lower BMD, though their overall prevalence and clinical significance remain under investigation [14,15].

The European Society for the Study of Coeliac Disease and the North American Society for the Study of Celiac Disease highlight the multifactorial nature of bone disease in CD, implicating not only malabsorption but also immune-mediated mechanisms and cytokine dysregulation [16]. While OPG and related pathways are promising as potential therapeutic targets, current management remains focused on a strict gluten-free diet, correction of nutritional deficiencies, and monitoring of bone health. No targeted therapies against OPG or RANKL are currently recommended specifically for CD-related bone loss, but these pathways remain under active investigation [11,12,16].

This study aimed to evaluate for the first time the relationship between osteoprotegerin levels, bone age, and growth parameters in CD children, and to explore the potential of osteoprotegerin as a biomarker for bone health in this population.

We predict that osteoprotegerin levels will correlate with growth and bone health parameters in CD children and may differ between newly diagnosed and long-term treated patients, reflecting distinct phases of bone metabolism and recovery. Specifically, we expect that OPG levels will be elevated in newly diagnosed cases and gradually normalize with adherence to a gluten-free diet. Further, we hypothesize that (*i*) newly diagnosed CD children (seropositive) would exhibit greater OPG than diet-compliant peers and controls; (*ii*) gluten withdrawal over 6 months would decrease OPG as inflammation subsides and (*iii*) OPG levels might predict delayed bone age, providing a noninvasive biomarker of skeletal health.

## 2. Methodology

### 2.1. Study Design and Setting

This was a case–control study conducted over three years (2018–2021) at the Department of Pediatrics, St. Marina University Hospital, Varna, Bulgaria. The study included two groups of children: those diagnosed with CD and age- and sex-matched controls without CD.

### 2.2. Participants

The study included a total of 146 children with an average age of 7.11 (±4.23) years, of whom 61 (42.4%) were males, categorized into three distinct groups:

Group A (TTG Positive at Inclusion): This group comprised 25 children with newly diagnosed celiac disease based on clinical presentation and positive tissue transglutaminase (TTG) serology. Not all participants in this group had yet started a gluten-free diet at the time of the first assessment.

Group B (TTG Negative at Inclusion): This group included 54 children with a previously confirmed diagnosis of celiac disease who had negative TTG serology at the time of inclusion, indicating compliance with and response to a gluten-free diet.

Group C (Control Group): This group consisted of 67 children without a diagnosis of celiac disease, matched by age and sex where possible. These children were recruited from the general pediatric population and had no clinical or laboratory evidence of celiac disease. Children in Group C were recruited consecutively from the general pediatric outpatient clinic at St. Marina University Hospital during routine healthy child visits. They had no history or symptoms suggestive of celiac disease and tested negative for celiac-specific serology (TTG-IgA), if tested, as part of differential diagnosis for other minor complaints.

All participants in Groups A and B underwent two assessments—at baseline and 6-month follow-up—while Group C underwent a single evaluation. Written informed consent was obtained from parents or legal guardians before enrollment.

### 2.3. Study Procedures

Children in the CD group were evaluated at two visits: at the time of diagnosis and six months after initiating a gluten-free diet (GFD), with or without vitamin D and calcium supplementation, depending on clinical indication and age-based dosing. The control group underwent a single evaluation.

### 2.4. Parameters Assessed

The following parameters were evaluated.

**Epidemiological:** Age and sex at both visits for the CD group and at a single time point for controls.

**Anthropometric:** Height (cm), weight (kg), and body mass index (BMI, kg/m^2^).

**Clinical:** Presence of gastrointestinal (GI) or extraintestinal symptoms and history of fractures.

**Laboratory:** CD-specific serology, osteocalcin (ng/mL, a marker of bone formation), and osteoprotegerin (OPG, pmol/L, a marker of bone resorption). These were measured at both visits in the CD group and once in controls.

**Instrumental:** Bone age—Bone age was evaluated using a left-hand and wrist radiograph interpreted according to the Greulich and Pyle atlas [17]. All radiographs were assessed by a pediatric radiologist blinded to the group allocation.

### 2.5. Inclusion Criteria

**Figure 1:**
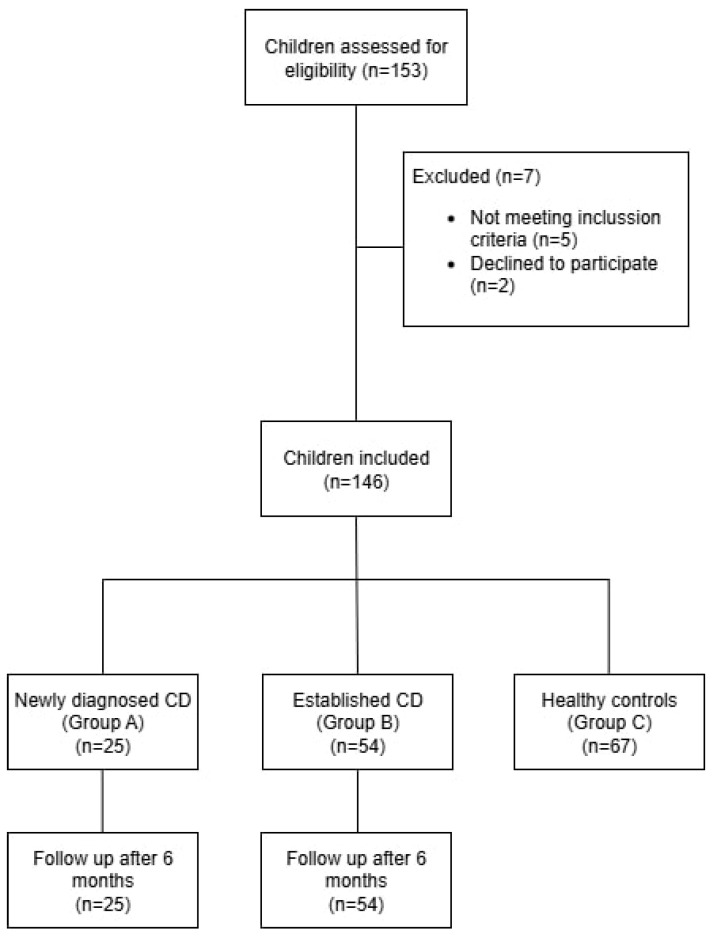
Flow Diagram of Participant Selection and Group Allocation.

Children were included in the CD group based on a confirmed diagnosis according to the ESPGHAN guidelines, including both newly diagnosed and previously diagnosed patients (Figure 1).

### 2.6. Data Collection

All participants underwent comprehensive history-taking, physical examination, and anthropometric assessment. Laboratory and radiological evaluations were conducted following standardized protocols. Data collection procedures complied with ethical standards and institutional guidelines. The study received ethical approval from the Medical Ethics Committee of the Medical University of Varna (Approval No. 76/09.08.2018). Written informed consent was obtained from the parents or legal guardians of all participants.

### 2.7. Statistical Analysis

All statistical analyses were conducted using the statistical package Jamovi 2.2.2. Descriptive statistics were used to summarize demographic, clinical, anthropometric, and biochemical data across the three groups: newly diagnosed CD patients (Group A), previously diagnosed CD patients (Group B), and healthy controls (Group C). Continuous variables were assessed for normality using the Shapiro–Wilk test and expressed as mean ± standard deviation (SD) for normally distributed data or median (interquartile range) for non-parametric data. Categorical variables were presented as frequencies and percentages.

Group comparisons for continuous variables were performed using one-way ANOVA with Bonferroni post hoc tests or Kruskal–Wallis tests, depending on the distribution. Differences in categorical variables were evaluated using Chi-square or Fisher’s exact test as appropriate.

Correlations between osteoprotegerin levels and clinical, anthropometric, or biochemical variables were analyzed using Spearman’s rank correlation coefficient (ρ). Paired-sample *t*-tests were employed to assess changes in osteoprotegerin levels over time within groups.

To assess the potential predictive role of osteoprotegerin for delayed bone age, binomial logistic regression models were constructed for each group at baseline and follow-up. Model fit was evaluated using McFadden’s pseudo-R^2^ and Akaike Information Criterion (AIC). A *p*-value of <0.05 was considered statistically significant unless otherwise specified. This was an exploratory study based on all eligible patients during the 3-year recruitment window. A post hoc power analysis using G*Power 3.1.9.7 indicated that the sample size (*n* = 146) was sufficient to detect a medium effect size (f = 0.25) with 80% power at α = 0.05 for comparisons among three groups (ANOVA).

### 2.8. Laboratory Analysis

Serum osteocalcin and osteoprotegerin were quantified using enzyme-linked immunosorbent assay (ELISA) kits (Human Osteocalcin ELISA Kit (ab270202), Manufacturer Abcam, Cambridge, UK), following manufacturer protocols. CD-specific serology was measured via quantitative IgA tissue transglutaminase (TTG-IgA) using a chemiluminescence immunoassay (CLIA; LIAISON^®^ tTG IgA, DiaSorin, Italy)). All samples were processed in the same certified clinical laboratory under blinded conditions.

## 3. Results

A total of 146 children were included in the study: 79 with celiac disease and 67 age- and sex-matched controls (Table 1). The celiac group was further subdivided into two cohorts—25 newly diagnosed children with positive TTG serology at inclusion (Group A) and 54 previously diagnosed children with negative serology, reflecting adherence to a gluten-free diet (Group B). The control group (Group C) included children without clinical or laboratory evidence of celiac disease. Across all participants, 83 (57.6%) were female and 61 (42.4%) were male. The mean age varied slightly between groups, with Group B being the oldest (8.15 ± 3.76 years), followed by Group A (7.28 ± 4.56 years), and Group C (6.20 ± 4.32 years), with the difference reaching statistical significance (*p* = 0.043). Post hoc analysis revealed that the difference was significant only between the control group and Group B (*p* = 0.033), while no statistically significant difference was observed between Groups A and B or A and C.

As shown in Table 1, height differed significantly between groups (*p* = 0.012), with Group B presenting the highest average values; however, post hoc analysis revealed that a significant difference was observed only between Group B and the control group (Group C). Although group differences in weight and BMI did not reach statistical significance, children with celiac disease—particularly those in Group A—more frequently fell below the fifth BMI centile. Gender distribution differed as well (*p* = 0.048), with a female predominance in Group B. Gastrointestinal symptoms were more prevalent among children with celiac disease than among controls, though this trend did not reach statistical significance (*p* = 0.082). They included abdominal pain (64.5%), bloating (32.9%), diarrhea (23.6%), and less frequently, nausea or vomiting. These were more prevalent in children with celiac disease compared to controls, though they did not reach statistical significance (*p* = 0.082).

No significant differences were observed in osteocalcin status across the groups, with most children demonstrating values within the normal range. Osteoprotegerin levels were highest in newly diagnosed patients (Group A), though the difference did not reach significance (*p* = 0.231), possibly reflecting a compensatory response to active inflammation or early bone turnover.

### 3.1. Correlations Between Osteoprotegerin and Clinical Parameters

Table 2 summarizes the significant Spearman’s correlation between osteoprotegerin and clinical or biochemical parameters across the three study groups. In Group A (TTG Positive), a strong positive correlation was observed between osteoprotegerin levels at baseline and follow-up (ρ = 0.458, *p* = 0.021) Notably, osteoprotegerin levels at the second visit were inversely correlated with both age (ρ = −0.634, *p* < 0.001) and height (ρ = −0.611, *p* = 0.001), suggesting a potential decline in bone resorption activity markers with advancing age and growth. Strong positive correlations were also seen between weight and height (ρ = 0.956), weight and age (ρ = 0.888), and weight and age at diagnosis (ρ = 0.727), consistent with expected growth patterns.

In Group B (TTG Negative), osteoprotegerin levels also showed a positive correlation across visits (ρ = 0.432, *p* = 0.001). Additionally, baseline osteoprotegerin was positively associated with serum iron (ρ = 0.327, *p* = 0.016), and inversely related to age at both visits (ρ = −0.284 and −0.334, respectively). Hemoglobin levels were negatively associated with age (ρ = −0.436, *p* = 0.001) and height (ρ = −0.461, *p* < 0.001), while osteoprotegerin levels showed a modest inverse correlation with weight (ρ = −0.270, *p* = 0.048).

Among the control children (Group C), osteoprotegerin levels were inversely correlated with age (ρ = −0.292, *p* = 0.018), weight (ρ = −0.331, *p* = 0.008), and height (ρ = −0.327, *p* = 0.007), reflecting a consistent pattern of declining bone resorption marker levels with physical growth.

### 3.2. Changes in Osteoprotegerin Levels over Time

Table 3 presents the results of the paired samples t-test comparing osteoprotegerin levels at baseline and at follow-up across three subgroups. In Group A, children who achieved negative serology by the second visit (*n* = 17), osteoprotegerin levels decreased from 5.82 ± 1.31 to 5.27 ± 1.35 pmol/L, with a trend toward statistical significance (*p* = 0.064), suggesting a possible reduction in bone turnover following dietary intervention.

In contrast, no significant change in osteoprotegerin was observed in children from Group A who remained serology-positive at the second visit (*p* = 0.553), nor in Group B children with sustained negative serology at both time points (*p* = 0.572), where levels remained relatively stable over time.

### 3.3. Predictive Value of Osteoprotegerin for Delayed Bone Age

Table 4 presents the results of binomial logistic regression analyses assessing the association between osteoprotegerin levels and delayed bone age across study groups. In Group A (newly diagnosed, TTG-positive), osteoprotegerin was not a significant predictor of delayed bone age at either the baseline or follow-up visit (*p* = 0.635 and *p* = 0.494, respectively). Model fit was poor, with low McFadden’s R^2^ values and high AICs, indicating limited explanatory value.

In Group B (previously diagnosed, TTG-negative), the model showed a statistically significant intercept (*p* = 0.016), and osteoprotegerin demonstrated a trend toward significance as a positive predictor of delayed bone age (Estimate = 0.470, *p* = 0.091), though it did not reach the threshold for statistical significance. At follow-up, osteoprotegerin was not significantly associated with delayed bone age (*p* = 0.617).

Interestingly, in the control group (Group C), higher osteoprotegerin levels were not significantly associated with delayed bone age (*p* = 0.283), but the intercept was statistically significant (*p* = 0.033), suggesting that other unmeasured factors may contribute to the presence of delayed bone age in this group.

Overall, the models suggest a limited but potentially emerging role for osteoprotegerin in predicting delayed bone age, particularly in children with longstanding celiac disease. However, effect sizes were small, and no associations reached full statistical significance across visits.

Notably, children with celiac disease in long-term remission (Group B) demonstrated similar regression patterns to healthy controls (Group C), with comparable model fit and lack of significant associations between osteoprotegerin and delayed bone age.

## 4. Discussion

To our knowledge, this is the first case–control study to longitudinally assess the relationship between OPG levels and radiologically confirmed bone age in pediatric CD. While OPG has been explored in adult populations, its dynamic profile in children during the transition from active to quiescent disease has not been reported before. This novel approach adds valuable insight into the search for non-invasive markers of skeletal health in pediatric autoimmune conditions.

### 4.1. Osteoprotegerin, Growth, and Nutrient Status: Insights into Bone Metabolism in Pediatric Celiac Disease

The observed correlations between osteoprotegerin and clinical characteristics across study groups provide further insight into the dynamic interplay between growth, inflammation, and bone metabolism in children with and without celiac disease. In newly diagnosed patients (Group A), the strong inverse associations between osteoprotegerin and both age and height at follow-up suggest that younger, shorter children—potentially at an earlier disease stage or with more active inflammation—may exhibit higher levels of bone resorption markers. The persistence of a positive correlation between baseline and follow-up osteoprotegerin also supports the idea of a stable individual bone resorption profile in the early post-diagnosis period, despite dietary intervention. This is consistent with other studies. For example, longitudinal studies in adult celiac disease patients demonstrate that, although a gluten-free diet (GFD) leads to improvements in bone mineral density (BMD) and reductions in bone turnover markers, individual profiles of osteoprotegerin (OPG) remain relatively stable over time, even after dietary intervention. This suggests that intrinsic factors—potentially genetic or immunological—may underlie a patient’s baseline bone resorption profile, and that these factors persist despite improvements in malabsorption and inflammation with GFD [10,11,12].

Specifically, persistently elevated OPG levels have been observed in celiac patients both before and after GFD initiation, and OPG correlates with bone turnover markers and BMD. The literature also notes that OPG may act as a compensatory response to increased bone resorption, but its levels do not fully normalize with dietary treatment, indicating a stable individual set point for bone metabolism [10,11,12]. This is consistent with the multifactorial pathophysiology of bone disease in celiac disease, as highlighted by the European Society for the Study of Coeliac Disease and the North American Society for the Study of Celiac Disease, which recognize that bone health in celiac disease is influenced by both reversible (diet-responsive) and stable (possibly genetic or immunological) factors [16].

The persistence of a positive correlation between baseline and follow-up osteoprotegerin supports the concept of a stable individual bone resorption profile in early post-diagnosis celiac disease, even with dietary intervention [10,11,12].

In previously diagnosed children with negative serology (Group B), similar age-related declines in osteoprotegerin were observed, accompanied by modest inverse associations with weight and a positive relationship with serum iron. These findings may reflect the complex recovery trajectory of bone metabolism during the long-term gluten-free diet, where improvements in nutritional status are gradual and may not fully normalize inflammatory markers. The negative associations between hemoglobin and both age and height may reflect residual or subclinical nutrient deficiencies that persist despite dietary treatment. Multiple studies demonstrate that, although most children with celiac disease experience improvement in hemoglobin and growth parameters after starting a gluten-free diet (GFD), a significant proportion continue to exhibit deficiencies in iron, ferritin, vitamin D, folate, and other micronutrients during long-term follow-up [3,18,19,20,21,22].

Specifically, iron deficiency and anemia are common at diagnosis and may persist in a subset of pediatric patients even after years on a GFD, with studies reporting persistent anemia in 3–4% of children after 1–10 years of dietary treatment [3,21,23]. Additionally, deficiencies in folate, vitamin D, and other micronutrients are frequently observed, and dietary intake assessments reveal that children with celiac disease often do not meet recommended intakes for these nutrients, even with nutritional education [18,22,24].

Growth impairment (lower height and weight z-scores) is common at diagnosis and improves with treatment, but normalization may be incomplete, particularly in those diagnosed at older ages or with more severe disease [4,25]. The persistence of suboptimal hemoglobin and growth parameters is thus consistent with ongoing or residual nutrient deficiencies, which may be due to incomplete mucosal healing, dietary imbalances, or both [3,18,19,20,21,22,25].

Osteoprotegerin has limited predictive value for determining bone age. In fact, despite plausible biology, OPG alone failed to predict delayed skeletal maturation. Serum OPG levels are influenced by multiple factors including age, sex hormones, renal function, and systemic diseases, and are not bone specific. While OPG levels generally increase with age in both men and women, studies consistently show that OPG does not correlate reliably with bone mineral density (BMD) or direct measures of bone turnover in healthy adults or in most disease states. For example, large cross-sectional studies demonstrate that OPG levels rise with age but do not predict BMD or bone microarchitecture with sufficient specificity or sensitivity to be clinically useful for bone age assessment [26,27,28,29,30]. The persistence of a positive correlation between baseline and follow-up osteoprotegerin supports the concept of a stable individual bone resorption profile in early post-diagnosis celiac disease, even with dietary intervention [10,11,12].

### 4.2. Osteoprotegerin as a Non-Conventional Inflammation Marker in Pediatric Celiac Disease

Although not a primary focus of this study, the elevated OPG levels observed at diagnosis may partly reflect systemic inflammation, consistent with previous reports in adult CD. However, we did not systematically assess inflammatory markers such as CRP or IL-6, so no conclusions can be drawn about OPG’s role as an inflammatory biomarker in this cohort. Osteoprotegerin is not routinely used as an inflammation marker in clinical practice. However, there is substantial evidence in the medical literature that osteoprotegerin (OPG) levels are associated with inflammatory states and correlate with established inflammatory markers such as C-reactive protein (CRP), interleukin-6 (IL-6), and erythrocyte sedimentation rate (ESR) in various conditions, including chronic kidney disease, infection-induced inflammation, and inflammatory bowel disease [31,32,33,34].

In our study, OPG is elevated at diagnosis which aligns with prior adult CD reports and its inverse relationship with height/age suggests that younger, smaller children, likely with more active mucosal injury, could upregulate OPG more strongly. Indeed, multiple studies have demonstrated that newly diagnosed, untreated adult coeliac disease patients exhibit increased serum osteoprotegerin (OPG) levels compared to healthy controls. This elevation is thought to represent a compensatory response to increased bone resorption and inflammation, as OPG acts as a decoy receptor for RANKL, inhibiting osteoclastogenesis and bone loss [10,11,12].

Studies have demonstrated that OPG concentrations increase in both acute and chronic inflammatory states and correlate with the severity of inflammation, but its sensitivity and specificity as an inflammation marker are inferior to established markers like CRP [32,34]. OPG is being investigated as a potential biomarker in specific disease contexts, such as inflammatory bowel disease and cardiovascular disease, but it is not currently recommended by any major clinical guidelines as a standard marker of inflammation [31,35].

### 4.3. Osteoprotegerin and Its Limited Role as a Predictor of Delayed Bone Age in Pediatric Celiac Disease

The longitudinal analysis of osteoprotegerin levels revealed that, while mean concentrations tended to decrease over time in newly diagnosed children who achieved serological remission (Group A, TTG-negative at follow-up), this change did not reach statistical significance (*p* = 0.064), though it approached the conventional threshold. This finding may reflect a gradual reduction in bone resorption activity following gluten withdrawal, consistent with the expected trajectory of mucosal healing and reduced systemic inflammation. In contrast, children who remained TTG-positive at follow-up (Group A non-responders) and those with longstanding serological remission (Group B) demonstrated stable osteoprotegerin levels over time, suggesting that bone remodeling markers plateau once dietary compliance is established or, alternatively, that osteoprotegerin reflects an individual set point in bone metabolism unaffected by short-term fluctuations in disease activity.

Logistic regression analyses did not identify osteoprotegerin as a significant independent predictor of delayed bone age in any group. However, the model for Group B showed a trend toward significance (*p* = 0.091), raising the possibility that in children with longstanding celiac disease, higher osteoprotegerin levels may be modestly associated with delayed skeletal maturation. The absence of a similar association in newly diagnosed or control children, along with generally poor model fit, highlights the multifactorial nature of bone age delay and the limited explanatory power of osteoprotegerin alone in this context. The significant model intercepts observed in Groups B and C suggest that other, unmeasured factors may play a more prominent role in determining bone maturation status, such as nutritional recovery, endocrine function, or chronic subclinical inflammation. Current evidence in the medical literature supports that osteoprotegerin (OPG) is associated with bone metabolism abnormalities in celiac disease, including altered bone mineral density (BMD) and increased bone turnover, but does not establish OPG as an independent predictor of delayed bone age in pediatric celiac disease. Studies in children and adults with celiac disease have shown that OPG levels are often elevated, likely as a compensatory response to increased bone resorption and that OPG correlates with BMD and bone turnover markers [10,11]. However, these studies do not demonstrate that OPG independently predicts delayed bone age when controlling for other variables.

Furthermore, systematic reviews and cohort studies in pediatric celiac disease populations focus on bone mineral content, bone mineral density, and risk factors such as BMI, vitamin D status, and disease duration, but do not identify OPG as an independent predictor of delayed bone age or bone mineral deficits in multivariate analyses [36,37,38]. Therefore, the statement is consistent with the current evidence base.

### 4.4. Limitations of Osteoprotegerin as a Biomarker for Skeletal Maturation in Celiac Disease

The results of the logistic regression analyses did not support osteoprotegerin as an independent predictor of delayed bone age in any of the study groups. In newly diagnosed children with celiac disease (Group A), OPG levels were not associated with bone age delay at either the initial or follow-up visit. The lack of predictive value, combined with low McFadden’s R^2^ and high AIC values, indicates limited model fit and suggests that other factors likely play a more substantial role in determining skeletal maturation in this group. This finding aligns with the previous literature, which has described elevated OPG in celiac disease as part of a compensatory mechanism for increased bone resorption [10,11], but not as a determinant of bone development outcomes.

In children with longstanding serological remission (Group B), OPG showed a trend toward significance as a positive predictor of delayed bone age at the first visit (*p* = 0.091), although the association did not meet conventional thresholds. The statistically significant model intercept (*p* = 0.016) suggests that other latent factors may contribute to the risk of delayed skeletal maturation in this subgroup. These might include persistent subclinical inflammation, micronutrient deficiencies, or incomplete mucosal healing—factors not directly captured by OPG levels alone. The absence of a similar trend at follow-up suggests instability in OPG’s predictive utility over time.

Interestingly, in the control group (Group C), OPG was also not significantly associated with delayed bone age. Yet, the significant intercept (*p* = 0.033) may indicate that bone age variability in healthy children is influenced by different, possibly developmental or nutritional, factors unrelated to inflammatory or autoimmune processes.

Overall, the models point to a limited but potentially nuanced role for osteoprotegerin in bone development outcomes among children with celiac disease. While OPG may reflect broader changes in bone remodeling and metabolic compensation, it does not appear to serve as a reliable standalone biomarker for predicting delayed bone age. Future studies integrating bone-specific biomarkers, inflammatory mediators, and imaging indices may help clarify the multifactorial pathways affecting skeletal maturation in this population.

Notably, children with celiac disease in long-term remission (Group B) demonstrated regression patterns similar to healthy controls (Group C), with comparable model fit and no significant associations between osteoprotegerin and delayed bone age. This suggests that well-managed celiac disease—characterized by normalized serology and clinical stability—may allow for bone maturation dynamics comparable to those of non-celiac children. These findings support the hypothesis that early diagnosis and strict adherence to a gluten-free diet can help mitigate long-term skeletal complications. At the same time, the lack of strong associations underscores the multifactorial nature of bone development in pediatric populations and highlights the need for broader biomarker panels and long-term follow-up to detect subtle variations in bone health recovery.

**Strengths and Limitations:** This study’s strengths include its prospective design, inclusion of both newly diagnosed and long-term treated children, and standardized assessment of bone age and biomarkers. Limitations include the relatively small sample size in subgroups, absence of cytokine profiling, lack of longitudinal data beyond six months, and absence of bone mineral density (BMD) measurements. A key limitation is the absence of direct measurements of proinflammatory cytokines such as IL-6 and IL-18, which have been shown to influence the OPG/RANKL axis. Future studies should incorporate these markers to elucidate inflammatory pathways in greater detail.

## 5. Conclusions

In this study, osteoprotegerin (OPG) was elevated in newly diagnosed children with celiac disease and showed expected correlations with age and growth parameters, but did not significantly change over time or independently predicted delayed bone age. While OPG may reflect underlying bone resorption activity, its limited variability and poor predictive value suggest it is not a reliable standalone marker for skeletal development. These findings highlight the multifactorial nature of bone health in pediatric celiac disease and the need for broader biomarker panels to guide monitoring and management.

## Figures and Tables

**Table 1:** Differences in Clinical, Biochemical, Anthropometric, and Demographic Characteristics by Group.

Variable	TTG Positive at Inclusion (Group A)	TTG Negative at Inclusion (Group B)	Controls (Group C)	*p*-Value
Age (years) ± SD	7.28 ± 4.56	8.15 ± 3.76	6.20 ± 4.32	0.043 *
Male, *n* (%)	14 (9.7%)	16(11%)	31(21.4%)	0.048 *
Weight-for-age centile ± SD	95.8 ± 8.9	84.1 ± 24.2	83.6 ± 24.7	0.169
Height-for-age centile ± SD	31.2 ± 35.4	55.6 ± 39.5	34.1 ± 36.7	0.012 *
BMI centile distribution, *n* (%)				0.102
<5th centile	8 (32.0%)	14 (25.9%)	20 (31.7%)	
5–85th centile	12 (48.0%)	34 (63.0%)	36 (57.1%)	
>85th centile	2 (8.0%)	6 (11.1%)	2 (3.2%)	
>95th centile	3 (12.0%)	0 (0.0%)	5 (7.9%)	
Gender, *n* (%)				0.048 *
Female	11 (44.0%)	38 (70.4%)	35 (53.0%)	
GIT symptoms, *n* (%)	23 (92.0%)	53 (98.1%)	58 (87.9%)	0.082
Abdominal Pain	45 (68.2%)	33 (61.1%)	14 (56.0%)	
Bloating	25 (37.9%)	17 (31.5%)	6 (24.0%)	
Diarrhea	20 (30.3%)	12 (22.2%)	4 (16.0%)	
Nausea or Vomiting	10 (15.2%)	5 (9.3%)	2 (8.0%)	
Yes				
Osteocalcin, *n* (%)				0.583
Normal	18 (72.0%)	44 (81.5%)	50 (75.8%)	
Low	7 (28.0%)	10 (18.5%)	17 (24.2%)	
Osteoprotegerin [pmol/L]	5.79 ± 2.91	5.08 ± 1.21	5.36 ± 1.47	0.231

Notes: * *p*-values from Fisher’s exact test: BMI (*n* = 142), Gender (*n* = 145), GIT-gastrointestinal tract symptoms (*n* = 145), Osteocalcin (*n* = 145). All group comparisons are shown. Statistically significant values are marked with * *p* < 0.05.

**Table 2:** Significant Correlations Between Osteoprotegerin and Clinical, Anthropometric, and Biochemical Parameters by Group.

Group	Variables	Spearman’s ρ	*p*-Value
Group A (TTG Positive)	Osteoprotegerin (Visit 1) − Osteoprotegerin (Visit 2)	0.458	0.021
Group A (TTG Positive)	Osteoprotegerin (Visit 2) − Age	−0.634	<0.001
Group A (TTG Positive)	Osteoprotegerin (Visit 2) − Height	−0.611	0.001
Group A (TTG Positive)	Weight − Height	0.956	<0.001
Group A (TTG Positive)	Weight − Age	0.888	<0.001
Group A (TTG Positive)	Weight − Age at diagnosis	0.727	<0.001
Group B (TTG Negative)	Osteoprotegerin (Visit 1) − Osteoprotegerin (Visit 2)	0.432	0.001
Group B (TTG Negative)	Iron − Osteoprotegerin (Visit 1)	0.327	0.016
Group B (TTG Negative)	Age − Osteoprotegerin (Visit 1)	−0.284	0.039
Group B (TTG Negative)	Age − Osteoprotegerin (Visit 2)	−0.334	0.016
Group B (TTG Negative)	Age − Hemoglobin	−0.436	0.001
Group B (TTG Negative)	Weight − Osteoprotegerin (Visit 1)	−0.270	0.048
Group B (TTG Negative)	Height − Hemoglobin	−0.461	<0.001
Group C (Control)	Osteoprotegerin − Age	−0.292	0.018
Group C (Control)	Osteoprotegerin − Weight	−0.331	0.008
Group C (Control)	Osteoprotegerin − Height	−0.327	0.007

**Table 3:** Paired Samples *t*-Test for Osteoprotegerin Levels at Baseline and Second Visit by Subgroup.

Subgroup	N	Osteoprotegerin Mean ± SD (Visit 1)	Osteoprotegerin Mean ± SD (Visit 2)	t Statistic	Df	*p*-Value
Group A with negative serology at Visit 2	17	5.82 ± 1.31	5.27 ± 1.35	1.99	16	0.064
Group A with positive serology at Visit 2	8	5.73 ± 5.02	4.45 ± 1.28	0.624	7	0.553
Group B with negative serology on Visit 1 and 2	53	5.02 ± 1.14	5.13 ± 1.29	−0.569	52	0.572

**Table 4:** Binomial Logistic Regression for Delayed Bone Age by Group and Visit.

Group	N	Outcome Variable	Predictor	Estimate	SE	Z	*p*-Value	R^2^ (McFadden)	AIC
Group A	25	Bone age	Osteoprotegerin [pmol/L]	−0.072	0.152	−0.475	0.635	0.0070	38.1
Group B	54	Bone age	Osteoprotegerin [pmol/L]	0.470	0.278	1.69	0.091	0.0524	58.2
Group C	67	Bone age	Osteoprotegerin [pmol/L]	0.214	0.199	1.07	0.283	0.0168	70.6
Group A	25	Second visit bone age	Second visit osteoprotegerin [pmol/L]	−0.308	0.450	−0.684	0.494	0.0228	25.5
Group B	54	Second visit bone age	Second visit osteoprotegerin [pmol/L]	0.178	0.355	0.501	0.617	0.0074	36.9

## Data Availability

The original contributions presented in the study are included in the article, further inquiries can be directed to the corresponding author.

## Abbreviations

AIC	Akaike Information Criterion
BMD	Bone Mineral Density
BMI	Body Mass Index
CD	Celiac Disease
CRP	C-Reactive Protein
ESPGHAN	European Society for Paediatric Gastroenterology, Hepatology and Nutrition
ESR	Erythrocyte Sedimentation Rate
GFD	Gluten-Free Diet
GI	Gastrointestinal
IL-6	Interleukin-6
IL-18	Interleukin-18
OPG	Osteoprotegerin
R^2^	McFadden’s Pseudo R-squared
RANKL	Receptor Activator of Nuclear Factor Kappa-B Ligand
SD	Standard Deviation
SE	Standard Error
TTG	Tissue Transglutaminase
